# Pathogenic effects of inhibition of mTORC1/STAT3 axis facilitates *Staphylococcus aureus*-induced pyroptosis in human macrophages

**DOI:** 10.1186/s12964-020-00677-9

**Published:** 2020-11-30

**Authors:** Ruiyuan Yao, Yuhao Chen, Huifang Hao, Zhixin Guo, Xiaoou Cheng, Yuze Ma, Qiang Ji, Xiaoru Yang, Yanfeng Wang, Xihe Li, Zhigang Wang

**Affiliations:** 1 State Key Laboratory of Reproductive Regulation and Breeding of Grassland Livestock, School of Life Sciences, Inner Mongolia University, Hohhot, 010070 China; 2 School of Life Sciences, Jining Normal University, Jining, 012000 China; 3 Research Center for Animal Genetic Resources of Mongolia Plateau, Inner Mongolia University, Hohhot, 010070 China

**Keywords:** Pyroptosis, *Staphylococcus aureus*, mTORC1, STAT3

## Abstract

**Background:**

Pyroptosis is a recently identified pathway of caspase-mediated cell death in response to microbes, lipopolysaccharide, or chemotherapy in certain types of cells. However, the mechanism of how pyroptosis is regulated is not well-established.

**Methods:**

Herein, the intracellular bacteria were detected by staining and laser confocal microscopy and TEM. Live/dead cell imaging assay was used to examine macrophage death. Western blot and immunohistochemical staining were used to examine the protein changes. IFA was used to identify typical budding vesicles of pyroptosis and the STAT3 nuclear localization. SEM was used to observe the morphological characteristics of pyroptosis. ELISA was used to detect the level of inflammatory cytokines. Pyroptosis was filmed in macrophages by LSCM.

**Results:**

*S. aureus* was internalized by human macrophages. Intracellular *S. aureus* induced macrophage death. *S. aureus* invasion increased the expression of NLRP3, Caspase1 (Casp-1 p20) and the accumulation of GSDMD-NT, GSDMD-NT pore structures, and the release of IL-1β and IL-18 in macrophages. Macrophages pyroptosis induced by *S. aureus* can be abrogated by blockage of *S. aureus* phagocytosis. The pyroptosic effect by *S. aureus* infection was promoted by either rapamycin or Stattic, a specific inhibitor for mTORC1 or STAT3. Inhibition of mTORC1 or STAT3 induced pyroptosis. mTORC1 regulated the pyroptosic gene expression through governing the nuclear localization of STAT3. mTORC1/STAT3 axis may play a regulatory role in pyroptosis within macrophages.

**Conclusions:**

*S. aureus* infection induces human macrophage pyroptosis, inhibition of mTORC1/STAT3 axis facilitates *S. aureus*-induced pyroptosis. mTORC1 and STAT3 are associated with pyroptosis. Our findings demonstrate a regulatory function of the mTORC1/STAT3 axis in macrophage pyroptosis, constituting a novel mechanism by which pyroptosis is regulated in macrophages.

**Video Abstract** Macrophages were infected with *S. aureus* for 3 h (MOI 25:1), and pyroptosis was filmed in macrophages by laser confocal microscopy. A representative field was recorded. Arrow indicates lysing dead cell.

## Background

Pyroptosis is a lytic form of caspase-dependent cell death, which encompasses caspase-1, -4, -5, and -11. Caspase-1 is activated by various canonical inflammasomes, and caspase-4/5/11 recognizes cytosolic bacterial lipopolysaccharide directly, both of which trigger pyroptosis [1]. In the canonical pathway, intracellular bacteria upregulate caspase-1, which then cleaves gasdermin D to produce N-terminal GSDMD fragment (GSDMD-NT) [2]. GSDMD-NT forms pores in the membrane that drive swelling and membrane rupture, and the cytokines IL-1β and IL-18 are released through GSDMD-NT pore structures [3]. Pyroptosis can defend against microbial challenges and endogenous threats by eliminating such compromised cells [4]. Many factors cause pyroptosis, including lipopolysaccharide, chemotherapy drugs, TNF-a, 17β-estradiol (E2), and bacteria [5–7].

As extracellular pathogens, certain types of bacteria can invade a variety of mammalian nonprofessional phagocytes and can be engulfed by professional phagocytes, such as neutrophils and monocytes, and survive in them [8, 9]. The uptake of bacteria by nonprofessional phagocytes is mediated by adhesins [10]. Fibronectin-binding proteins, a major type of adhesin, mediate the internalization of adherent bacteria [11]. Professional phagocytic cells, such as neutrophils and macrophages, are designed to engulf microorganisms and clear debris—a process that can be divided conceptually into formation of the phagosome and subsequent evolution into a degradative compartment [12]. The monocyte-derived macrophages (MDMs) are separated into two specific phenotypes, classically activated macrophages (M1) and alternatively activated ones (M2) [13], Macrophages differentiate into M1 induced by IFN-γ and LPS, and the M1macrophages participate in the removal of pathogens during infection, induce M1 phenotype polarization which have main roles in host defense against various microbial pathogens and increased tumoricidal activity [14, 15]. Therefore, M1-polarized human MDMs were used as a cell model for macrophages function [16, 17].

Several pathogens can invade cells and survive intracellularly for various periods [18]. The success of intracellular pathogens is attributed to their ability to inhibit phagocytosis by preventing opsonophagocytosis and blocking specific signaling pathways [19], such as avoiding delivery to the lysosome and release into the cytoplasm and arresting phagosome maturation, creating an optimal niche for replication [20].

*Staphylococcus aureus*, a gram-positive bacterium and human pathogen that causes a wide range of illnesses, from skin infections to severe pneumonia and sepsis, can invade cells and trigger pyroptosis in human macrophages [7]. However, the mechanism by which it is regulated in professional phagocytes is unknown.

Mammalian target of rapamycin complex 1 (mTORC1) is a central coordinator in eukaryotic cells with regard to cell growth and metabolism with environmental inputs [21], autophagy, apoptosis, necroptosis, and other forms of cell death [22]. mTORC1 regulates autophagy by inhibiting ULK and the nuclear translocation of transcription factor EB (TFEB) [23, 24] and suppresses apoptosis in pterygium by controlling Beclin1-dependent autophagy by targeting Bcl-2 [25]. Among the intrinsic forms of cell death, pyroptosis has received increased attention recently, by the function of mTORC1 in pyroptosis is unkonwn.

Signal transducer and activator of transcription 3 (STAT3) is a latent transcription factor that mediates extracellular signals, such as those from cytokines and growth factors [26]. STAT3 plays an important role in programmed cell death, and inhibition of STAT3 leads directly to apoptosis [27]. In addition, autophagy is governed by STAT3 activation by upregulating or downregulating essential autophagy genes [28]. However, the relationship between STAT3 and pyroptosis has not been reported. Moreover, mTOR has been implicated in the regulation of STAT3 activation [29], and mTORC1 stimulates STAT3 to restrain proinflammatory responses [30]. Nevertheless, the regulatory function of mTORC1 in the expression of pyroptosic genes via STAT3 and pyroptosis is unknown.

Pyroptosic macrophages are important in the defense against microbial infections, removing pathogens and rendering them susceptible to phagocytosis and killing by a secondary phagocyte [31, 32]. As mentioned previously, mTORC1 and STAT3 are associated with autophagy and apoptosis. Thus, in this study, we hypothesized that mTORC1/STAT3 axis mediates pyroptosis via regulating expression of pyroptosic genes in human macrophages. We examined whether mTORC1 signaling regulates *S. aureus*-induced pyroptosis and whether the inhibition of the mTORC1/STAT3 axis causes pyroptosis in human macrophages. Collectively, our study demonstrates that mTORC1 and STAT3 have critical functions in the regulation of *S. aureus*-induced pyroptosis and that inactivation of the mTORC1/STAT3 axis causes pyroptosis in human macrophages. Our results implicate a novel function of the mTORC1/STAT3 axis in regulating cell death and provide insights into the mechanism by which pyroptosis is governed in professional phagocytic cells.

## Methods

### Reagents and antibodies

The anti-GSDMD (Cat# ab215191), anti-caspase-1 (Cat# ab1872), anti-S6 (Cat# ab184551), anti-4EBP1 (Cat# ab2606), anti-p-mTOR (Ser2448) (Cat# ab32028), anti-mTOR (Cat# ab10926), goat anti-rabbit (Cat# ab136817), and anti-mouse (Cat# ab205719) secondary antibodies were purchased from Abcam (Abcam plc 330 Cambridge Science Park, Cambridge, UK.) The anti-p-S6 (Ser240/244) (Cat# 5346s), anti-STAT3 (Cat# 4904s) anti-p-4EBP1 (Thr37/46) (Cat# 2855s), and anti-p-STAT3 (Cat# 4113s) were purchased from Cell Signaling Technology (Cell Signaling Technology, Inc., Beverley, MA, USA). The anti-GSDMD (Cat# abs128820) was purchased from Absin (Absin Co., Ltd. Shanghai, China). The goat FITC-conjugated anti-rabbit IgG (Cat# 115-095-003) and FITC-conjugated anti-mouse IgG (Cat# 115-095-146) were purchased from Jackson (Jackson ImmunoResearch Laboratories, Inc., West Grove, PA, USA). The anti-β-actin (Cat# A5441) was purchased from Sigma (Sigma-Aldrich, Inc. St. Louis, MO, USA).

LPS (Cat# L2630), Stattic (Cat# S7947), CFSE (Cat# 21888), and PMA (Cat# P1585) were purchased from Sigma-Aldrich, Inc. (St. Louis, MO, USA). Rapamycin (Cat# 53123-88-9) was purchased from (Gene Operation, Ann Arbor, MI, USA). IFN-γ (Cat# 121527) was purchased from PEPROTECH (PEPROTECH Inc., Rocky Hill, USA). β-mercaptoethanol (Cat# M8211) was obtained from Solarbio (Solarbio Science and Technology, Co., Ltd. Beijing, China). Dil (Cat# KGMP002) was purchased from KeyGEN (KeyGEN BioTECH, Co., Ltd. Jiangsu, China). DAPI (Cat# C1005) was acquired from Beyotime (Beyotime Biotechnology, Co., Ltd. Shanghai, China). Alexa Fluor® 594 Phalloidin (Cat# A12381) was purchased from Invitrogen (Invitrogen, Carlsbad, New Mexico, USA). Cytochalasin B (Cat# HY-16928) was purchased from MCE (MedChemExpress, New Jersey, Monmouth, USA).

### Cell culture

Human myeloid leukemia mononuclear (THP-1) cells and THP-1-derived macrophages were cultured in RPMI-1640 medium (Hyclone Laboratories, Inc. Logan, UT, USA) that was supplemented with 10% fetal bovine serum (BI Biological Industries, Beit Haemek Israel), 100 U/mL penicillin G, 100 mg/mL streptomycin (Sigma-Aldrich, Inc. St. Louis, MO, USA), and 0.1% β-mercaptoethanol (Solarbio Science and Technology, Co., Ltd. Beijing, China). To induce the classical (M1) polarization program, the medium was replaced with fresh medium without 10% fetal bovine serum and with PMA (100 ng/mL) for 24 h and then supplemented with 2.5 ng/mL IFN-γ and 100 ng/mL LPS for 24 h. Cells were cultured at 37 °C in humidified air with 5% CO_2_. Macrophages were infected by *S. aureus* (ATCC25923) at a multiplicity of infection (MOI) of 25:1 (bacteria to macrophages).

### Spread plate method

Macrophages were infected with *S. aureus* for 3 h at an MOI of 25, and the extracellular bacteria were killed and lysed by antibiotics and lysozyme for 2 h. Monolayer macrophages were lysed, and the number of intracellular bacteria was determined by spread plate method.

### Bacteria and macrophage staining

The macrophages were seeded onto a slide and incubated overnight. Bacteria (*S. aureus*) were washed with PBS and then incubated with CFSE (5(6)-carboxyfluorescein diacetate *N*-succinimidyl ester) at 4 °C for 15 min. The stained bacteria were centrifuged for 10 min at 3000×*g* at 4 °C 3 times. Macrophages were infected by the stained bacteria at an MOI of 25, washed with PBS 3 times, and then fixed with 4% paraformaldehyde for 20 min. After being treated with 1% Triton X-100 for 5 min, the macrophages were stained with Alexa Fluor® 594 Phalloidin for 1 h in the dark, washed with PBS 3 times, and counterstained with 100 μL DAPI for 3 min to assess the nuclear morphology. Finally, the slide was mounted with glycerinum for examination under a laser scanning confocal microscope (LSCM) (NIKON AIR, Nikon Corp., Tokyo, Japan).

### Immunofluorescence assay (IFA)

The cells were seeded onto a slide, incubated overnight, washed with PBS, and fixed with 4% paraformaldehyde for 15 min. After treatment with 1% Triton X-100 for 10 min, the cells were blocked with 1% BSA for 1 h, stained with GSDMD/Caspase-1 primary antibody overnight at 4 °C, and incubated with FITC-labeled goat anti-rabbit/mouse IgG for 1 h at room temperature. Dil was added at 37 °C for 12 min to stain the membrane, and nuclear was stained with 100 μL DAPI for 3 min. Finally, the slide was mounted with glycerinum and examined under an LSCM (NIKON AIR, Nikon Corp., Tokyo, Japan).

### TEM and SEM

Macrophages were infected by bacteria (*S. aureus*) for 3 h at an MOI of 25, and the extracellular bacteria were killed and lysed by antibiotics and lysozyme for 2 h. The infected macrophages were washed with PBS 3 times centrifuged for 10 min at 3000×*g* at 4 °C, and fixed with 2.5% glutaraldehyde overnight. Finally, the cells were embedded in 4% AGAR and fixed with 2.5% glutaraldehyde overnight. The samples were examined by TEM (Hitachi HT7700, Hitachi, Ltd., Tokyo, Japan) to detect intracellular bacteria. For SEM, macrophages were seeded onto a slide and incubated overnight. The macrophages were infected by bacteria or treated with rapamycin, washed with PBS, and fixed with 2.5% glutaraldehyde for 4 h. After being air-dried, the slides were examined by SEM (Hitachi S-4800, Hitachi, Ltd., Tokyo, Japan) to detect GSDMD-NT pores on the cytomembrane.

### Western blot

Macrophages were washed with cold PBS and lysed in cell lysis buffer. The lysis buffer comprised 50 mM Tris (pH 7.4), 150 mM NaCl, 1% Triton X-100, 1% sodium deoxycholate, 0.1% SDS, 1% PMSF, and phosphatase inhibitors. Equal amounts (40 μg) of protein were electrophoresed on 10% (w/v) sodium dodecyl sulfate–polyacrylamide gels, transferred to polyvinylidene fluoride membranes, and incubated with the primary antibody. Peroxidase-conjugated secondary antibody and enhanced chemiluminescence (ECL) reagent were used to detect the signals with the Western Blotting System (GE Healthcare Bio-Sciences, Pittsburgh, PA, USA).

### Immunohistochemical staining

Cells were seeded onto slides, incubated overnight, washed with PBS, and fixed with 4% paraformaldehyde for 15 min. For immunohistochemical detection of proteins (1000× magnification), antigens were detected using UltraSensitive™ S-P Immunohistochemistry hypersensitive kits (mouse/rabbit) (MXB Biotechnology Co. Ltd., Fujian, China) with hematoxylin (MXB Biotechnology Co. Ltd., Fujian, China) for counterstaining. A biotin-streptavidin peroxidase-based method was used to detect the primary antibodies.

### ELISA

Levels of IL-1β and IL-18 in the supernatant was quantified by enzyme linked immunosorbent assay (ELISA) using NeoBioscience ELISA kits (Neobioscience Technology Co. Ltd., Shenzhen, China). The amount of culture media and intracellular α-hemolysin were measured via standard Mbbiolog ELISA kit (Mbbiology Biological Technology Co. Ltd., Jiangsu, China) according to the manufacturer’s guidelines. Absorbance at 450 nm and 630 nm was read on a Varioskan Flash Multimode Reader (Thermo Fisher Scientific, Pittsburgh, PA, U.S.A.). All measurements were performed in triplicate, and the mean value of 3 independent measurements was used for statistical analysis.

### Confocal microscopy and light microscopy

Mounted IFA slides were observed under an LSCM (NIKON AIR, Nikon Corp., Tokyo, Japan). The macrophages phenotype, IHC slides, and large bubbles on the macrophages were observed under a light microscope (NIKON Eclipse80i, Nikon Corp., Tokyo, Japan).

### qRT-PCR analysis

Quantitative real-time PCR (qPCR) was performed to determine the levels of *CASP1*, and *GSDMD* in macrophages in the treatment and control groups. For the bacterial infection, macrophages were infected with *S. aureus* for 3 h at an MOI of 25, and the extracellular bacteria were killed and lysed by antibiotics and lysozyme for 2 h. For the inhibitor treatment, macrophages were treated with 100 nM rapamycin for 6 h or 100 μM Stattic for 1 h, and the total RNA was extracted from untreated and treated cells. Total RNA was prepared with the RNAiso Plus reagent according the manufacturer's instructions (Takara Co. Ltd., Dalian China). Briefly, the cells were washed with PBS and lysed in RNAiso Plus, and chloroform was added to the cell lysates for homogenization. The top aqueous layer was transferred to a new tube after centrifugation, and isopropanol was added to the supernatant and mixed well. Total RNA was precipitated by centrifugation, and the pellet was dissolved in RNase-free water. RNA quantities over 600 ng/μL and a purity of 1.90–2.0, based on the 260/280 ratio, were used to synthesize cDNA.

mRNA was reverse-transcribed with oligo (dT)_12–18_ primer using the AMV 1st Strand cDNA Synthesis Kit (Takara Co. Ltd., Dalian China). *ACTB* was selected as the internal control gene. The primer sequences were as follows (5′–3′):

- *CASP1*: forward, CGACAAGGTCCTGAAGGAGAAGAG
  reverse, CGTGTGCGGCTTGACTTGTCCATT
- *GSDMD*: forward, TGTACGTGGTGACTGAGGTGCTGC
  reverse, CTGAGGGGATGGTGACCGTCTTCT
- *ACTB*: forward, TCACCAACTGGGACGACAT
  reverse, GCACAGCCTGGATAGCAAC

The KAPA SYBP® FAST qPCR Kit Optimized for LightCycler® 480 (KAPA BIOSYSTEMS, Inc., Boston, MA, U.S.A.) was used for the PCR according to the manufacturer’s instructions. The program comprised an initial denaturation step at 95 °C for 5 min; 40 cycles of 95 °C for 5 s, 54 °C for 30 s, and 72 °C for 20 s; and a final extension of 72 °C for 10 min. Three technical replicates were run in each experiment. 2^−ΔΔCT^ values were calculated to determine expression levels, and the qPCR results were analyzed by student’s t-test to compare the expression between untreated and treated groups. Three independent experiments were performed.

### Caspase-1 activity assay

The caspase-1 activity was measured using a Caspase1 Activity Assay kit (Beijing solarbio science and technology co., ltd. Beijing, China). According to the manufacturer’s instructions, about 50 μg protein from cells was mixed with synthetic tetrapeptide Ac-YVAD-pNA and incubated at 37 °C for 2 h. A standard curve was prepared using the *p*NA standard. The absorbance was determined at 405 nm with a 96-well plate reader and the caspase1 activity was normalized for total proteins of cell lysates.

### Live/dead cell imaging assay

It is performed using the Live & Dead® Viability/Cytotoxicity Assay Kit (US Everbright, Inc., Suzhou, China). Macrophages were infected with *S. aureus* and incubated with 2 μM Calcein AM and 4 μM PI solution. Incubation at room temperature for 15–20 min and observe labeled cells under Live cell Imaging System (NIKON TI-E, Nikon Corp., Tokyo, Japan.)

### Phagocytosis assays

Macrophages were treated with 100 nM rapamycin for 6 h or 10 μg/mL Cytochalasin B for 30 min, respectively, cells and *S. aureus* were co-cultured at an MOI of 25 for 3 h to enable phagocytosis. Phagocytosis was stopped by placing on ice and washing the macrophages cultures twice to remove non-phagocytosed bacteria. The extracellular bacteria were killed and lysed by antibiotics and lysozyme. Monolayer macrophages were lysed, and the number of intracellular bacteria was determined by spread plate method.

### Statistical analyses

Statistical analyses were conducted using SPSS PASW Statistics for Windows, v18.0 (SPSS Inc.: Chicago, IL, USA). Data were analyzed using standard parametric statistics and one-way ANOVA, followed by Tukey’s method. Data are expressed as mean ± SD. The results are presented as the average of at least 3 independent experiments. Statistical significance was accepted when *p* ≤ 0.05.

## Results

### Phagocytosis of *S. aureus* by macrophages

M1-polarized human MDMs increase their caspase-1 activity, release of IL-1β, and loss of cell membrane integrity compared with the M2 program [7, 33]. To characterize *S. aureus*-induced pyroptosis in macrophages, THP-1 cells were differentiated into macrophages in culture with 100 ng/mL PMA (phorbol myristate acetate) for 24 h. Then, the macrophages were treated in differentiation medium with 2.5 ng/mL IFN-γ and 100 ng/mL LPS for 24 h to transform them into M1-polarized macrophages (Additional file 1: Figure S1a). The M1 macrophages released more TFN-α and IL-1β than the Mϕ phenotype (Additional file 1: Figure S1b), indicating that M1-polarized macrophages were successfully induced by IFN-γ and LPS.

**Fig. 1:**
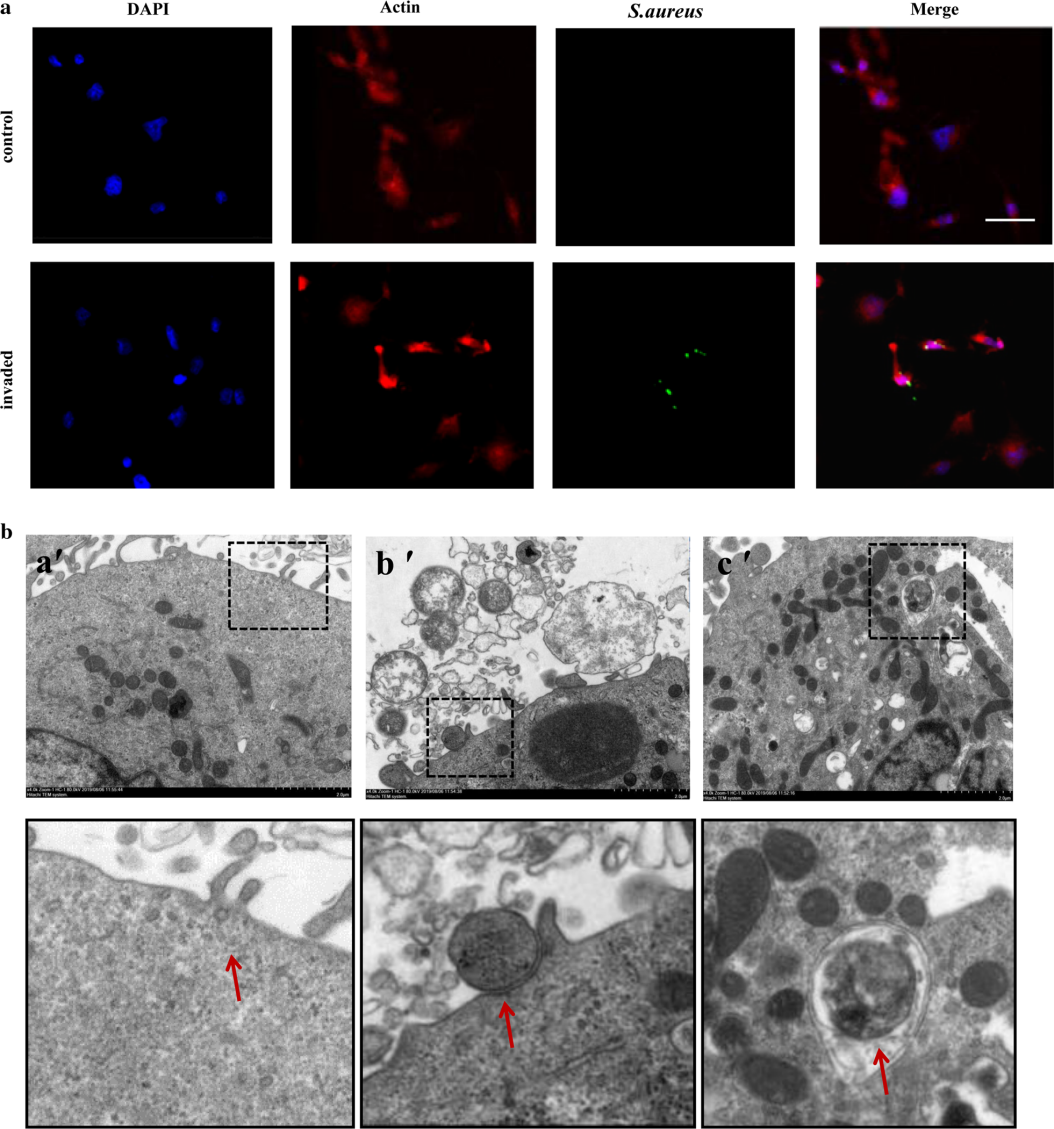
*Staphylococcus aureus* invades macrophages. *S. aureus* infected macrophages for 3 h, and then, the macrophages were cultured in medium supplemented with antibiotics and lysozyme to kill and lyse the extracellular bacteria. The intracellular bacteria were detected by staining and laser confocal microscopy and TEM. **a** The intracellular *S. aureus* (green) stained with CFSE was observed by laser confocal microscopy; macrophages nuclei were co-stained with DAPI (blue), and actin was stained with phalloidin (red). Scale bars represent 50 μm. **b** Bacteria were attached to the cell membrane and engulfed, based on micrographs obtained by TEM; several important observations are magnified. Red arrows indicate *S. aureus*. Scale bars represent 2 μm. **a′** Control, non-infected human macrophages. **b′** The bacteria were located predominantly in the plasma membrane; macrophages were allowed to engulf bacteria and initiate phagocytosis. **c′** *S. aureus* was engulfed by macrophages via the formation of typical phagocytic cups

To measure the internalization of *S. aureus* by macrophages, we infected M1 phenotype macrophages with *S. aureus* for 3 h and then killed and lysed the extracellular bacteria with antibiotics and lysozyme. The bacteria were evaluated intracellularly and extracellularly by bacterial colony count—7.5 ± 0.12 × 10^3^ CFU/mL were counted in whole-cell lysates, whereas extracellular bacteria were not found in the culture medium (Additional file 2: Table S1). Further, intracellularly stained bacteria were observed under a laser scanning confocal microscope (LSCM) (Fig. 1a), and under a transmission electron microscope (TEM), *S. aureus* adhered to the cell membrane and invaded the macrophages (Fig. 1b). These data indicate that *S. aureus* was internalized by the macrophages.

### Intracellular *S. aureus* induces pyroptosis of macrophages

**Fig. 2:**
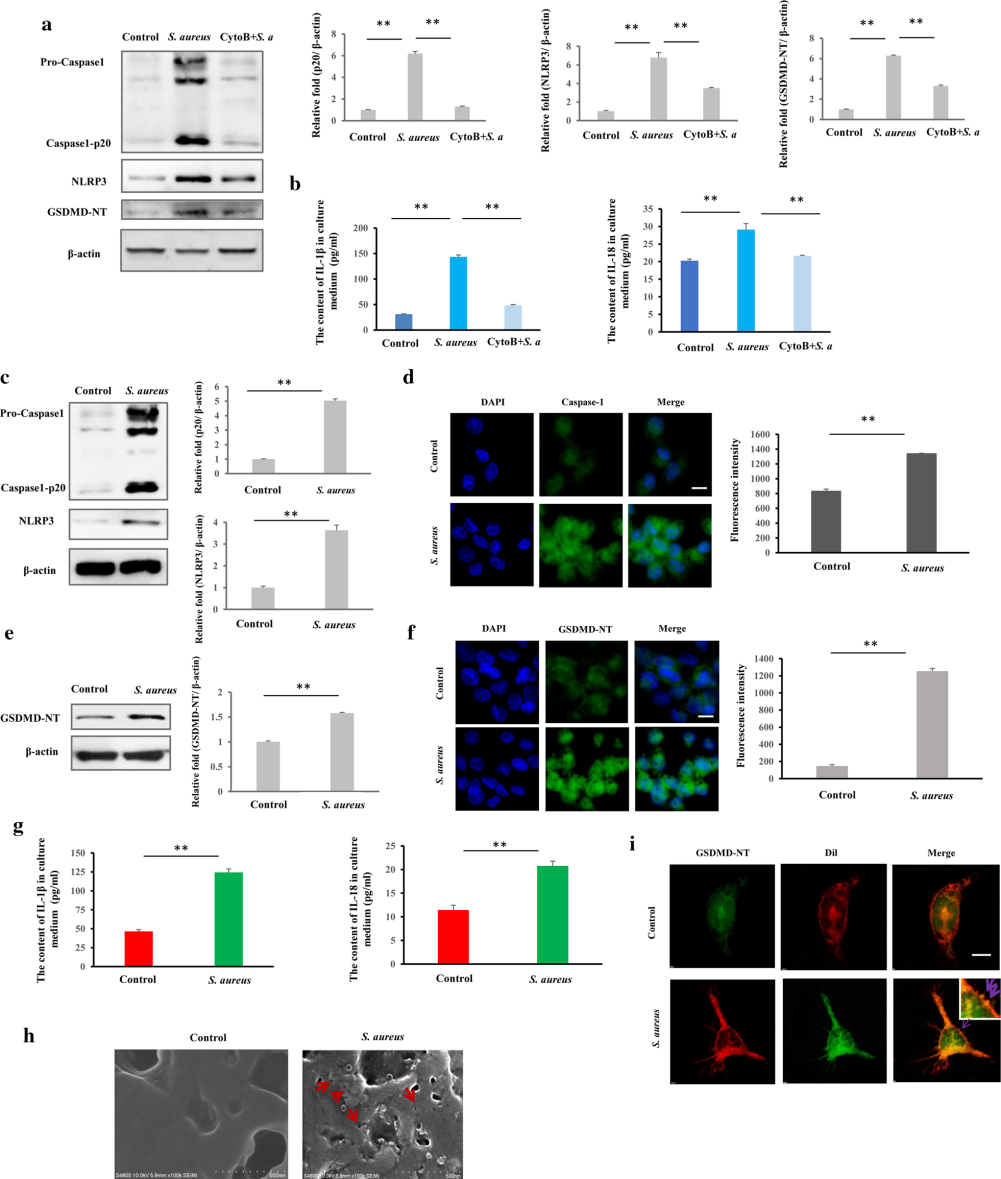
*Staphylococcus aureus* triggers pyroptosis in human macrophages. Cells were infected with *S. aureus* for 3 h (MOI 25:1), and the pyroptotic characteristics were examined, including pyroptosic protein markers, inflammatory cytokines release, and morphology. **a** Expression of pyroptosis-related proteins in response to *S. aureus* invasion and Cytochalasin B was assessed by western blot. NLRP3, caspase1-p20 and GSDMD-NT proteins were less prominent than *S. aureus* invasion after Cytochalasin B treated. **b** The levels of IL-18 and IL-1β were quantified by an ELISA. The levels of IL-18 and IL-1β were less prominent than *S. aureus* invasion after Cytochalasin B treated. **c** Western blot analysis showed *S. aureus* enhanced NLRP3 and caspase1-p20 expression in infected macrophages. **d** Immunofluorescence assay showed greater caspase 1 expression in infected macrophages than in the control. Representative confocal microscopy images of caspase 1 expression (Green) in cells that were co-stained with DAPI (blue). Scale bars represent 10 μm. **e** GSDMD-NT expression was examined in *S. aureus*-infected macrophages and controls by western blot. *S. aureus* invasion induced greater GSDMD-NT expression in infected macrophages. **f** Immunofluorescence assay showed greater GSDMD-NT expression in infected macrophages than in the control. Representative confocal microscopy images of GSDMD-NT expression (green) in cells that were co-stained with DAPI (blue). Scale bars represent 10 μm. **g** IL-18 and IL-1β levels were determined by ELISA. *S. aureus* invasion induced more release of IL-18 and IL-1β in infected macrophages. **h** Scanning electron microscopy of GSDMD-NT pores on plasma membrane in *S. aureus*-infected macrophages. Red arrow indicates GSDMD-NT pore. Scale bar, 500 nm. **i** GSDMD-NT (green) in cells co-stained with Dil (red) as a membrane marker. Representative confocal microscopy images of *S. aureus*-infected macrophages and control cells by immunofluorescence assay. The purple arrows indicate the necks of budding vesicles, indicating shedding of wounded plasma membrane in *S. aureus*-infected macrophages. Scale bars represent 1 μm. The resolved bands were quantified using Gel-Pro Analyzer 4.0 (Media Cybernetics, Inc., Rockville, MD, USA). Fluorescence intensity of the immunofluorescent was measured by imaging analysis software (NIS-Elements Viewer, Nikon Instruments Inc. Shanghai, China). *p < 0.05; **p < 0.01. n = 3 independent experiments. Error bar indicates SD

Considering *S. aureus* can induce NLRP3-mediated signaling triggering caspase-1 activation and programmed necrosis through the production of a pore-forming toxin in macrophages [7, 34], to eliminate the possibility of pore-forming toxin induces NLRP3 inflammasome-dependent pyroptosis during *S. aureus* infection period, α-hemolysin as the most prominent [35], we first evaluated the levels of α-hemolysin (Hla) in cell medium and in macrophages. The results showed that α-hemolysin was not detectable both in cell medium and macrophages (Additional file 3: Figure S2a), indicating α-hemolysin was not accumulated during the 3 h infection period. To determine whether inhibition of phagocytosis was specifically restricted to the engulfment of *S. aureus* and has effect on pyroptosis, macrophages were treated with Cytochalasin B, an inbibitor of phagocytosis [36], and intracellular bacteria were detected. The results showed that the number of intracellular bacteria were statistically decreased in Cytochalasin B treated cells (Additional file 3: Figure S2b). Furthermore, the activation of NLRP3 and Caspase-1, the expression of GSDMD-NT, IL-1β, and IL-18 triggered by *S. aureus* were abrogated by Cytochalasin B (Fig. 2a, b). These data indicate that blockage of *S. aureus* phagocytosis could abrogate the pyroptosis induction.

Intracellular bacteria may lead to cell pyroptosis, which is accompanied by caspase-1 activation, following the generation of GSDMD-NT fragment to form membrane pores and the release of IL-18 and IL-1β [37]. To determine whether pyroptosis is induced by *S. aureus* infection in human macrophages, we first measured the expression of several markers by western blot, immunofluorescence, and immunohistochemistry staining. NLRP3, Caspase-1 (Casp-1 p20) (Fig. 2c, d) and GSDMD-NT (Fig. 2e, f and Figure S2c), were upregulated in cells that harboured intracellular *S. aureus*. By ELISA, compared with control, IL-18 and IL-1β levels were significantly increased (*p* < 0.05) in the medium of cells that contained bacteria (Fig. 2g).

Next, to examine the morphological characteristics of macrophages that were infected by *S. aureus*, a scanning electron microscope (SEM) was used to observe the GSDMD-NT pore structures on the plasma membrane. Pore structures with ~ 30-nm diameters were observed (Fig. 2h). Moreover, when the cell membrane was damaged, the exocytic patching mechanism was initiated to repair the membranes by removing the pores in cells with few gasdermin pores [38]. We examined the typical budding vesicles that act during the shedding of damaged plasma membranes using LSCM, interesting, we found that budding vesicles were present in macrophages with intracellular *S. aureus* but not control cells (Fig. 2i). Further, once GSDMD pores form at levels that exceed the cell’s compensatory abilities, cells begin to swell, with characteristic large bubbles that protrude from the plasma membrane [39]. Thus, a light microscope was used to examine the bubbles that formed on the macrophages—large bubbles were observed on cells with intracellular *S. aureus* versus control cells (Additional file 3: Figure S2d). By video, cell swelling with characteristic large bubbles and lytic cells were seen (Video Abstract). Notably, by live/dead cell imaging assay, *S. aureus* was found to induce macrophage death (Additional file 4: Video 1). Taken together, these results suggest that intracellular *S. aureus* induces pyroptosis in human macrophages.

### Rapamycin promotes *S. aureus*-induced pyroptosis in macrophages

**Fig. 3:**
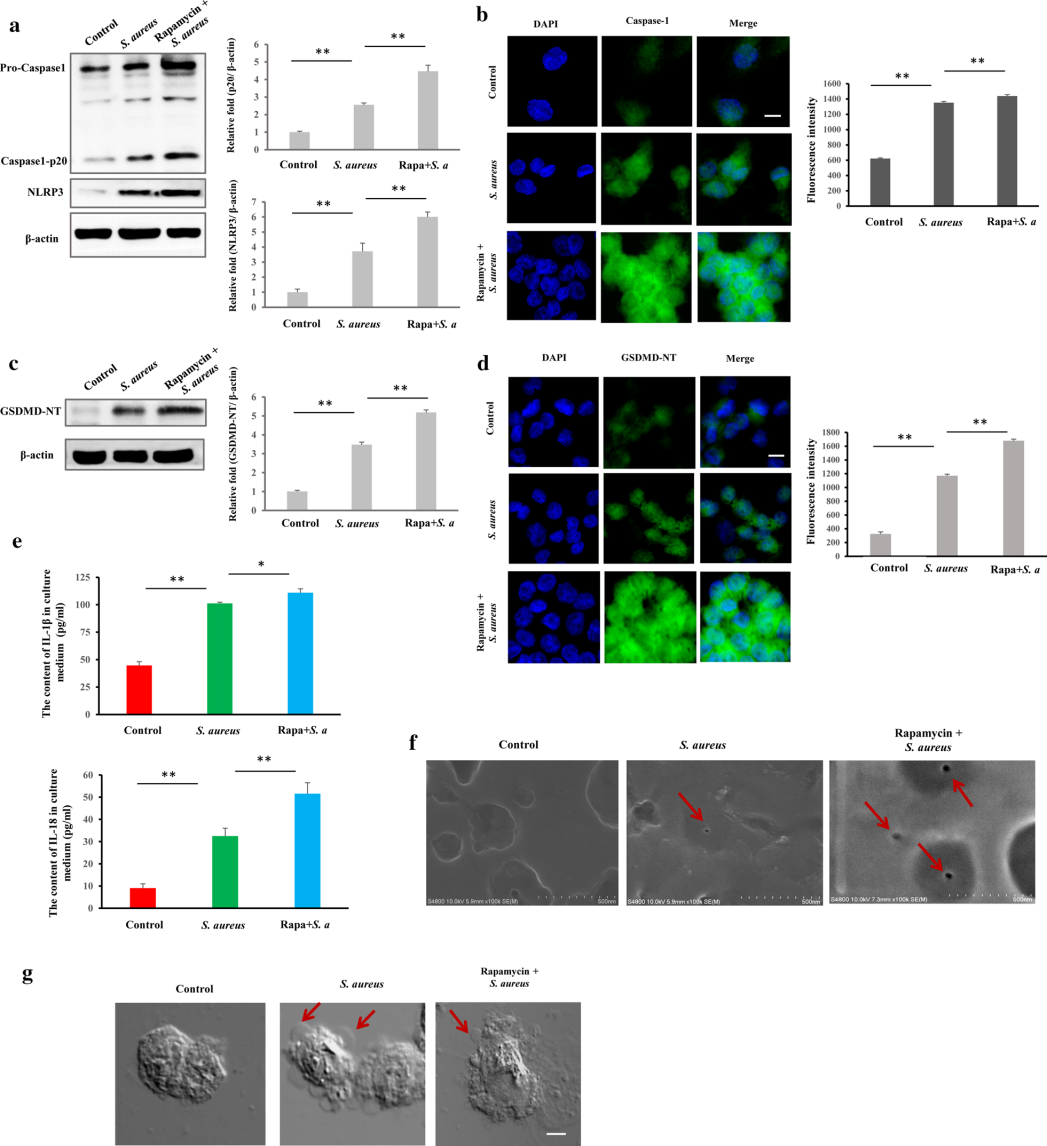
Rapamycin promotes *Staphylococcus aureus*-induced pyroptosis in macrophages. Macrophages were pretreated with rapamycin (100 nM) for 6 h and then infected with *S. aureus* for 3 h. The pyroptotic characteristics were determined, including pyroptosic protein markers, inflammatory cytokine release, and morphology. **(a** Western blot analysis of *S. aureus*-induced NLRP3 and caspase1-p20 expression. **b** Immunofluorescence assay of *S. aureus*-induced caspase 1 expression. Representative confocal microscopy images of caspase 1 (green) in cells that were co-stained with DAPI (blue). Scale bars represent 10 μm. **c** GSDMD-NT expression by western blot. **d** GSDMD-NT expression by immunofluorescence assay. Representative confocal microscopy images of GSDMD-NT (green) in cells that were co-stained with DAPI (blue). Scale bars represent 10 μm. **e** Macrophages were pretreated with rapamycin for 6 h and then infected with *S. aureus* for 3 h. The levels of IL-18 and IL-1β in cell culture medium were determined by ELISA*.* **f** GSDMD-NT pores on plasma membrane by scanning electron microscopy. Red arrow indicates GSDMD-NT pore. Scale bar, 500 nm. **g** Large bubbles by light microscopy, as indicated by red arrows in pyroptotic cells. Scale bars represent 1 μm. The resolved bands were quantified using Gel-Pro Analyzer 4.0 (Media Cybernetics, Inc., Rockville, MD, USA). Fluorescence intensity of the immunofluorescent was measured by imaging analysis software (NIS-Elements Viewer, Nikon Instruments Inc. Shanghai, China). *p < 0.05; **p < 0.01. n = 3 independent experiments. Error bar indicates SD

As discussed, mTORC1 regulates autophagy and apoptosis. Thus, we hypothesized that *S. aureus*-induced pyroptosis is associated with mTORC1 signaling. We treated *S. aureus-*invaded cells with rapamycin and then examined the expression of caspase-1 (Casp-1 p20), NLRP3 and GSDMD-NT by western blot, immunofluorescence, and immunohistochemistry staining and the levels of IL-18 IL-1β in the cell medium by ELISA. Rapamycin upregulated the *S. aureus-*induced expression of caspase-1 (Casp-1 p20) and NLRP3 (Fig. 3a, b), GSDMD-NT (Fig. 3c, d and Additional file 5: Figure S3a) and the release of IL-18 and IL-1β Fig. 3e) (*p* < 0.01). Further, rapamycin promoted the formation of *S. aureus-*induced GSDMD-NT membrane pores (Fig. 3f) and bubbles (Fig. 3g). Moreover, to assess the effect of rapamycin on macrophage uptake of *S. aureus*, macrophages that had engulfed bacteria were analysed by bacterial colony count. Notably, rapamycin did not influence the ability of macrophages to phagocytose bacteria (Additional file 5: Figure S3b). These data suggest that rapamycin accelerates *S. aureus-*induced pyroptosis in macrophages and that mTORC1 is involved in pyroptosis.

### mTORC1 inhibition causes macrophage pyroptosis

**Fig. 4:**
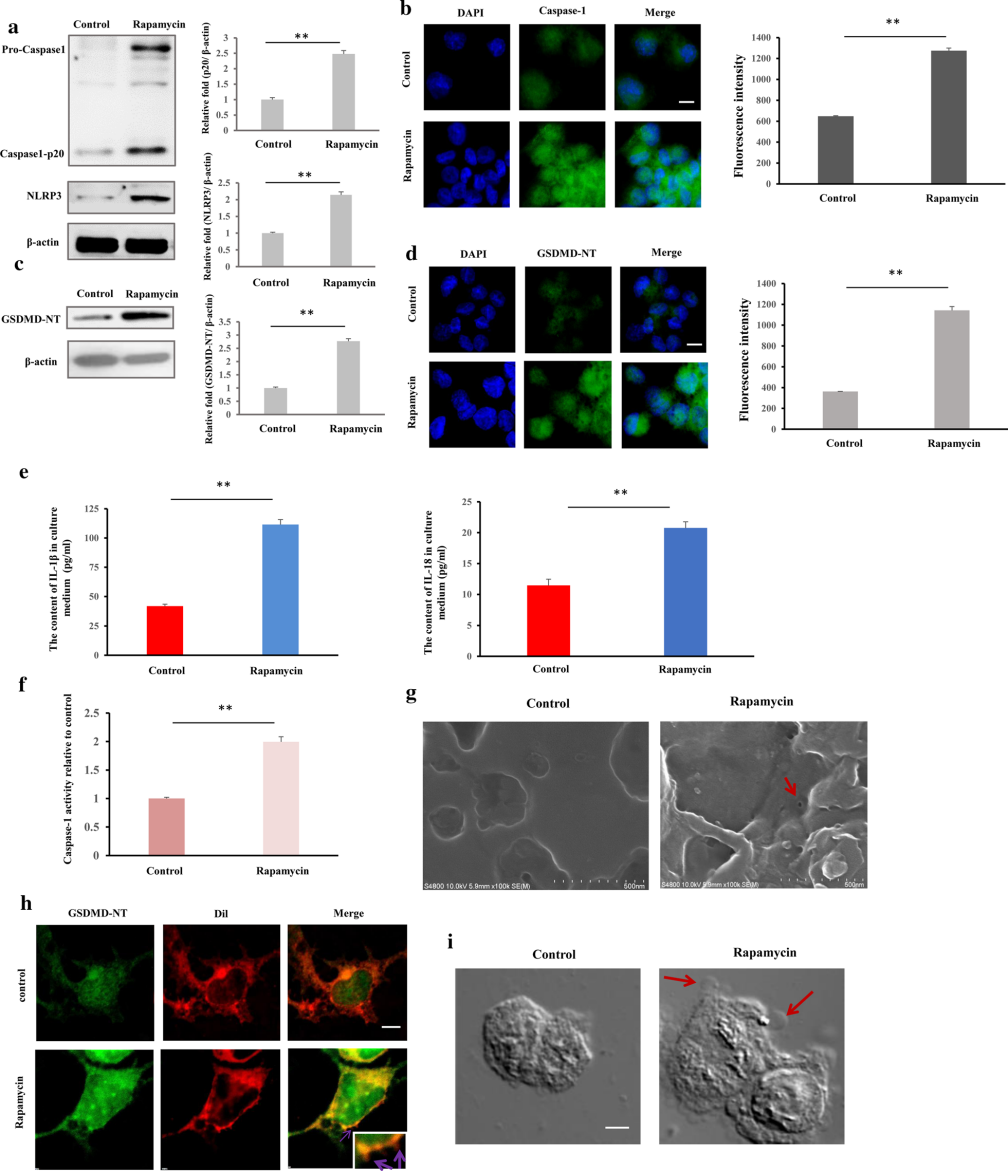
Rapamycin causes pyroptosis in macrophages. Macrophages were treated with rapamycin (100 nM) for 6 h, and the pyroptotic characteristics were examined. **a** Western blot of NLRP3 and caspase1-p20 expression. **b** Immunofluorescence assay of caspase 1 expression. Representative confocal microscopy images of caspase 1 expression (green) in cells that were co-stained with DAPI (blue). Scale bars represent 10 μm. **c** Rapamycin upregulates GSDMD-NT expression. **d** Rapamycin upregulates GSDMD-NT expression in cells. Representative confocal microscopy images of caspase 1 expression (green) in cells that were co-stained with DAPI (blue) by immunofluorescence assay. Scale bars represent 10 μm. **e** Rapamycin increases the levels of IL-18 and IL-1β in cell culture medium. **f** Whole cell lysates were extracted from macrophages, Caspase-1 activity was determined by colorimetric assay and induced by rapamycin treatments. **g** Scanning electron microscopy of GSDMD-NT pores on plasma membrane in rapamycin-treated macrophages. Red arrow indicates GSDMD-NT pore. Scale bar, 500 nm. **h** GSDMD-NT (green) in cells co-stained with Dil (red) as membrane marker. Representative confocal microscopy images of rapamycin-treated macrophages and control cells by immunofluorescence assay. The purple arrows indicate the necks of budding vesicles in rapamycin-treated macrophages. Scale bars represent 1 μm. **i** Large bubbles by light microscopy, indicated by red arrows in pyroptotic cells. Scale bars represent 1 μm. The resolved bands were quantified using Gel-Pro Analyzer 4.0 (Media Cybernetics, Inc., Rockville, MD, USA). Fluorescence intensity of the immunofluorescent was measured by imaging analysis software (NIS-Elements Viewer, Nikon Instruments Inc. Shanghai, China). *p < 0.05; **p < 0.01. n = 3 independent experiments. Error bar indicates SD

Based on the data above, we speculated that rapamycin induces pyroptosis in macrophages. To this end, we treated cells with 100 nM rapamycin for 6 h. We first examined mTORC1 signaling and found the phosphorylation of mTOR, S6, and 4EBP1 decreased (Additional file 6: Figure S4a), indicating that the mTORC1 pathway is inhibited by rapamycin. By western blot, immunofluorescence, and immunohistochemistry, NLRP3, caspase-1 (Casp-1 p20), (Fig. 4a, b) and GSDMD-NT (Fig. 4c, d and Additional file 6: Fig. S4b) were upregulated by rapamycin, and the release of IL-18 and IL-1β was enhanced by rapamycin (Fig. 4e) (*p* < 0.01). Meanwhile, using a colorimetric assay to assess caspase-1 activity during rapamycin treatment, we found that caspase-1 activity was significantly increased (Fig. 4f). As in *S. aureus-*invaded cells, rapamycin induced the formation of GSDMD-NT membrane pores (Fig. 4g) by SEM. Budding vesicles from the plasma membrane were observed under an LSCM (Fig. 4h), acting as a membrane damage repair mechanism and appearing early during pyroptosis. Bubbles were also observed under a light microscope (Fig. 4i). Further, by video, cell swelling with characteristic large bubbles and lytic cells were seen (Additional file 7: Video 2). These data indicate that inhibition of mTORC1 causes pyroptosis in macrophages and that mTORC1 signaling has inhibitory effects on pyroptosis in macrophages.

### mTORC1 regulates the expression of *CASP1* and *GSDMD* through STAT3 in macrophages

The experiments above demonstrated that rapamycin increased the protein expression levels of caspase-1 (Casp-1 p20) and GSDMD-NT; previous study has shown that STAT3 is associated with apoptosis and autophagy and thus [28], we hypothesized that mTORC1 may regulate these proteins through STAT3 in macrophages.

**Fig. 5:**
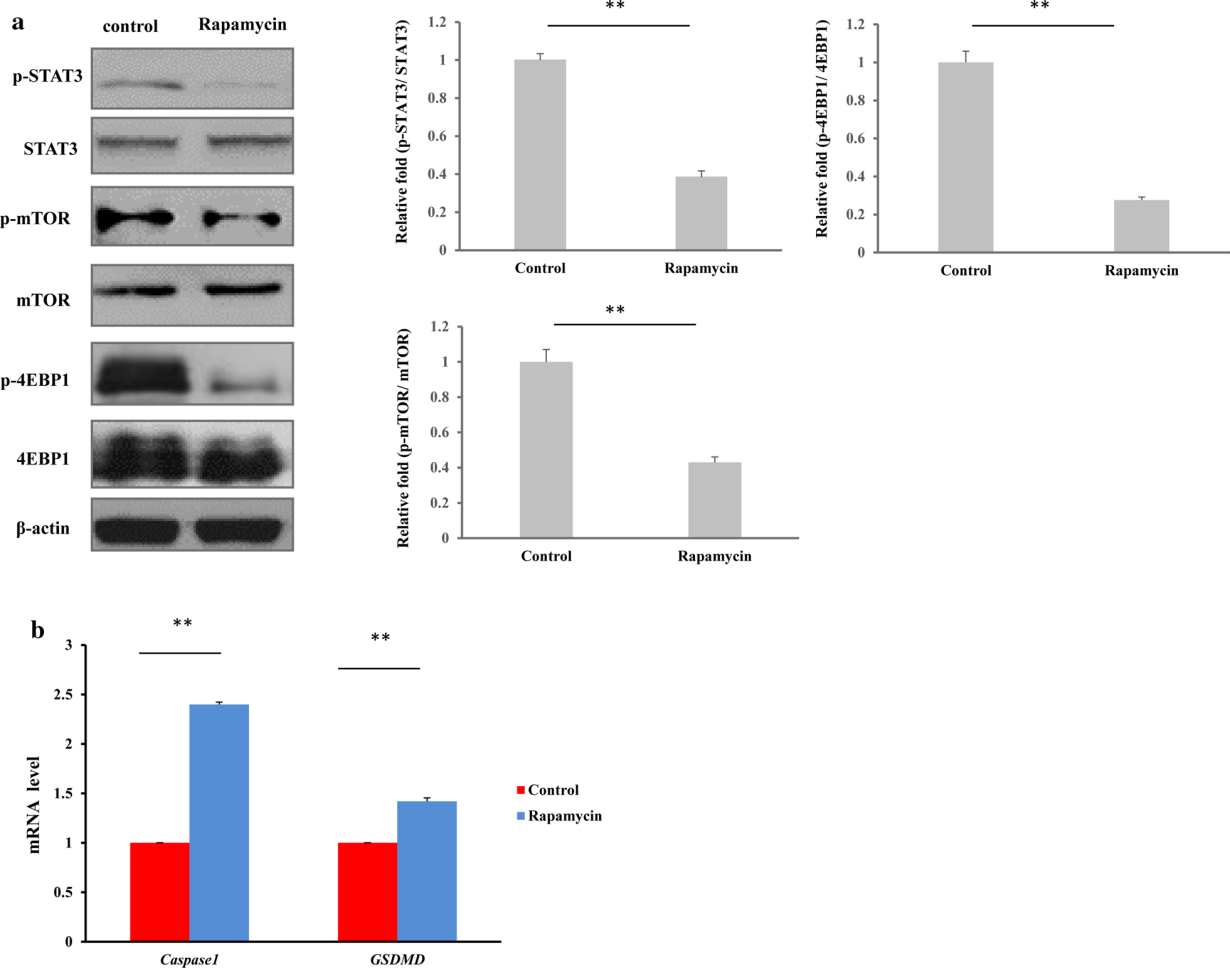
mTORC1 regulates the expression of *CASP1* and *GSDMD* through STAT3 in macrophages. Macrophages were treated with rapamycin (100 nM) for 6 h, and the activity of mTORC1 and STAT3 and the expression of pyroptosic genes were examined. **a** Western blot analysis of mTORC1 activation and STAT3 activation. Phosphorylation of STAT3 was inhibited. **b** Expression of *CASP1* and *GSDMD*, mRNA upregulated by rapamycin. The resolved bands were quantified using Gel-Pro Analyzer 4.0 (Media Cybernetics, Inc., Rockville, MD, USA). *p < 0.05; **p < 0.01. n = 3 independent experiments. Error bar indicates SD

To determine whether mTORC1 regulates the expression of *CASP1* and *GSDMD* via STAT3, we first treated macrophages with 100 nM rapamycin for 6 h and found that repamycin reduced the phosphorylation of STAT3, indicating STAT3 activation was inhibited (Fig. 5a). Then, we measured the mRNA levels of these pyroptosic genes by qRT-PCR, demonstrating that rapamycin upregulates *CASP1* and *GSDMD* (Fig. 5b) (*p* < 0.01). These data suggest that mTORC1 regulates the expression of pyroptosic genes, likely through STAT3 in macrophages.

### STAT3 inhibition causes macrophage pyroptosis through upregulations of CASP1 and GSDMD

**Fig. 6:**
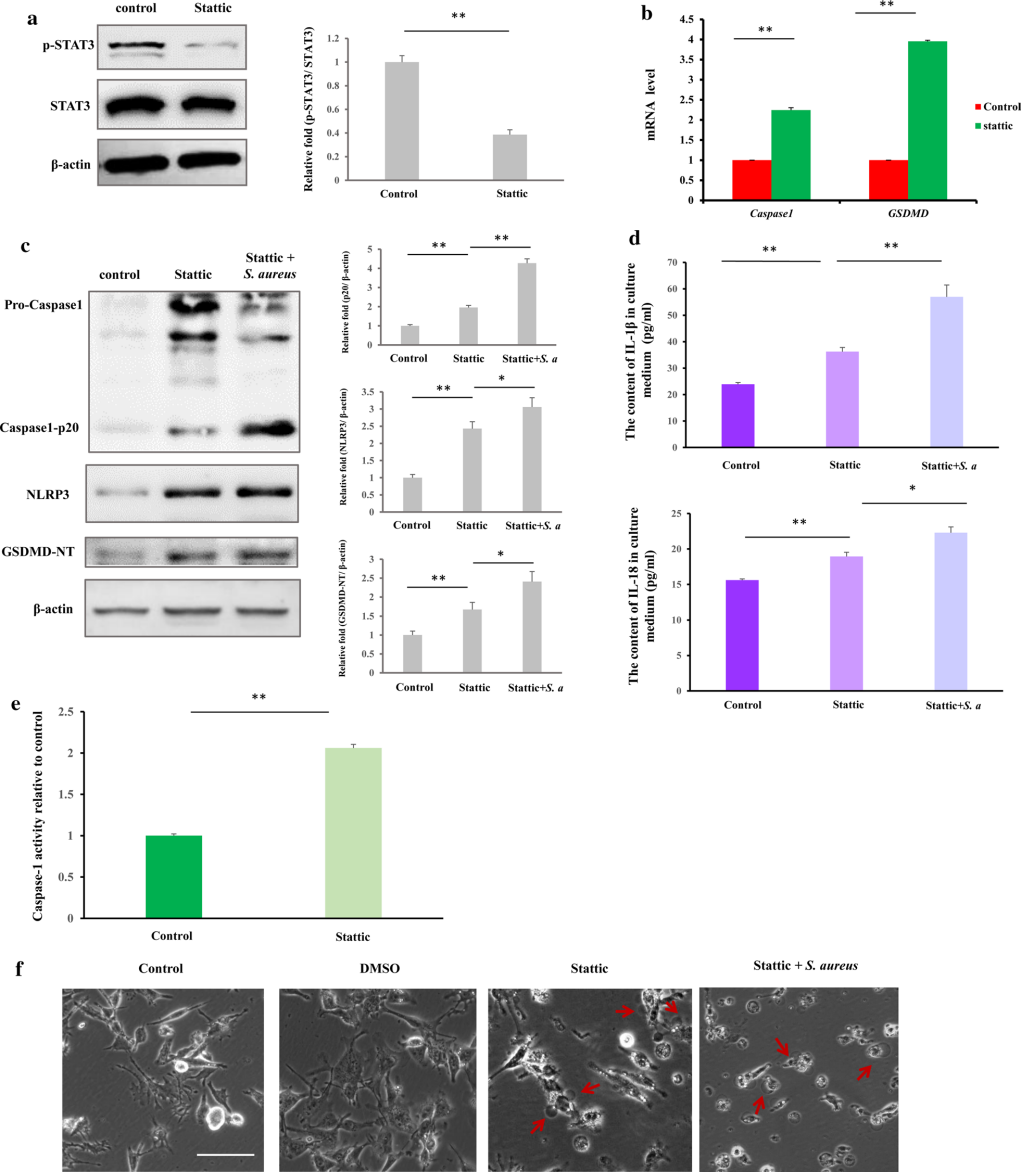
Inhibition of STAT3 by Stattic upregulates expression of pyroptosic genes and causes pyroptosis in macrophages. Macrophages were treated with Stattic (100 μM) for 1 h to inhibite STAT3 activation. The expression of pyroptosic genes and pyroptosic protein markers and the release of inflammatory cytokines were examined. **a** Stattic inhibits STAT3 phosphorylation in cells. **b** Stattic enhances the expression of *CASP1* and *GSDMD* mRNA in macrophages. **c** Stattic enhances NLRP3, caspase1-p20 and GSDMD expression, pyroptosis-related proteins were severally elevated in *S. aureus* infected cells which pretreated with Stattic. **d** Stattic increases the production of IL-18 and IL-1β in cell culture medium. **e** Caspase-1 activity was enhanced by Stattic treatment. **f** Stattic triggers pyroptosis. Red arrows indicate large bubbles in pyroptic cells. Scale bars represent 100 μm. The resolved bands were quantified using Gel-Pro Analyzer 4.0 (Media Cybernetics, Inc., Rockville, MD, USA). *p < 0.05; **p < 0.01. n = 3 independent experiments. Error bar indicates SD

To determine whether STAT3 affects the expression of *CASP1*, and *GSDMD*, we treated cells with Stattic, a selective inhibitor of STAT3 activation and dimerization. Stattic inhibited STAT3 phosphorylation in macrophages (Fig. 6a). Stattic increased the mRNA levels of *CASP1* and *GSDMD* (Fig. 6b), indicating that their expression was governed by STAT3 in macrophages. Further, cells were pretreated with Stattic and exposed to *S. aureus* produced more NLRP3, Casp-1 p20 and GSDMD-NT comparing to only Stattic treated and untreated cells (Fig. 6c). By ELISA, the levels IL-18 and IL-1β in the cell medium increased in treated cells (Fig. 6d) (*p* < 0.01). In addition, Stattic treatment significantly induced caspase-1 activation (Fig. 6e). These data suggest that inhibition of STAT3 induces pyroptosis in macrophages. By video, pseudopod contracting and cell rounding (Additional file 8: Video 3), cell swelling with characteristic large bubbles and lytic cells were observed (Fig. 6f and Additional file 9: Video 4). Thus, STAT3 is critical in pyroptosis, controlling the expression of pyroptosic genes in human macrophages.

### Inactive mTORC1 prevents the nuclear localization of STAT3 in macrophages

**Fig. 7:**
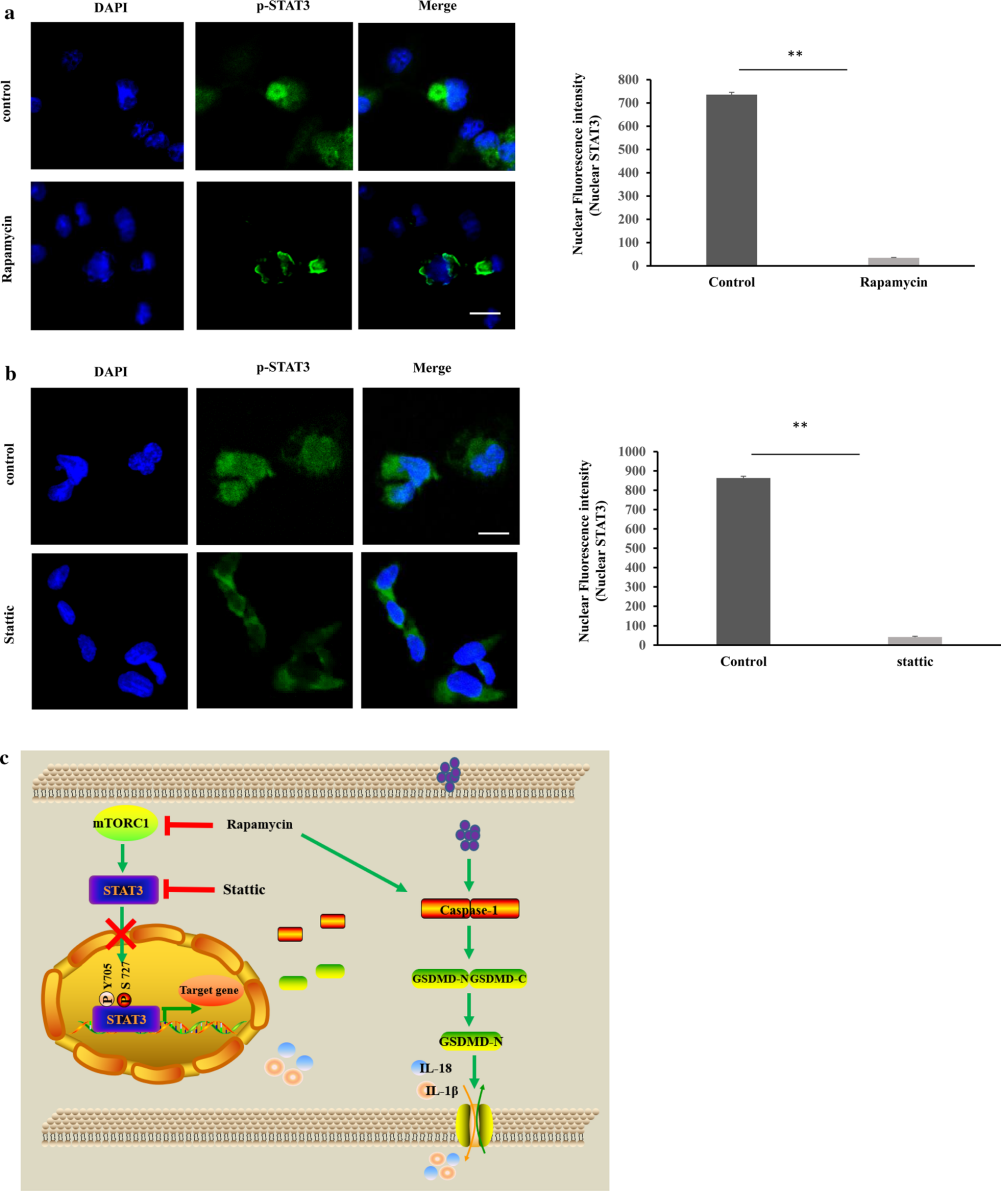
Inactive mTORC1 prevents the nuclear localization of STAT3 in macrophages. **a** Nuclear localization of STAT3 was prevented in cells treated with rapamycin. Scale bars represent 10 μm. The nuclear fluorescence intensity of STAT3 was shown. **b** Nuclear localization of STAT3 was prevented in cells treated with Stattic. Scale bars represent 10 μm. The fluorescence intensity of nuclear STAT3 was graphed. **c** The model of mTORC1 regulating pyroptosis through nuclear localization of STAT3 and pyroptosic gene expression in human macrophage. STAT3 is likely a negative regulator of pyroptosic gene expression. mTORC1 inhibition by rapamycin or STAT3 inhibition by Stattic prevents the nuclear localization of STAT3, upregulates pyrotosic gene expression, triggering human macrophage pyroptosis

Based on the results above, we reasoned that mTORC1 governs the nuclear localization of STAT3 to regulate the expression of pyroptosic genes. To this end, immunofluorescence was performed to confirm whether mTORC1 promotes the nuclear localization of STAT3. Cells were treated with rapamycin or Stattic as a positive control. The nuclear localization of STAT3 was prevented in cells that were treated with either compound, compared with control (Fig. 7a, b), indicating that the nuclear localization of STAT3 is controlled by mTORC1. These results demonstrate that mTORC1 signaling negatively regulates human macrophage pyroptosis by regulating the nuclear localization of STAT3 and pyroptosic gene expression (Fig. 7c).

## Discussion

During cell death, cell-intrinsic effector functions are coordinated to restrict infection and resolve innate and adaptive immune responses. The most widely used classification of cell death had consisted of 2 types: apoptosis and necrosis [40], but in recent years, necroptosis and pyroptosis have been confirmed [41]. Apoptotic cells retain plasma membrane integrity, versus pyroptosis or necroptosis is the rupture of cell lytic, which weakens the integrity of the plasma membrane and allows the influx of extracellular ions and fluid, leading to cell swelling [42].

In lytic pyroptosis, variety of cytokines are released, rendering it an inflammatory event [43]. Pyroptosis is a form of caspase-mediated cell death, and its regulatory mechanism is poorly understood. Studies have demonstrated that rapamycin induces apoptosis in peritoneal carcinomatosis [44] and necrosis in cardiac cells [45], and mTORC1 negatively regulates autophagy by inhibiting ULK [24]. mTORC1 is a central regulator of apoptosis, necrosis, and autophagy. However, the regulatory function of mTORC1 in pyroptosis has not been reported. In this study, we found that *S. aureus* induces pyroptosis in human macrophages and that the inhibition of mTORC1 by rapamycin promotes *S. aureus*-induced pyroptosis. These data suggest that mTORC1 regulates pyroptosis in macrophages.

Pyroptosis was initially observed in macrophages that were infected with *Salmonella typhimurium* or *Shigella flexneri* [46, 47]. Cytosol-invasive bacteria, such as *Listeria monocytogenes,* induce pyroptosis [48]. Of note, *S. aureus* pore-forming toxins (PFTs) induce NLRP3-mediated signaling, triggering caspase-1 activation and pyroptosis in human and mouse monocytic cells [49]. In our study, the effect of PFTs on pyroptosis have been eliminated and pyroptosis can be abrogated by blockage of *S. aureus* phagocytosis. These data indicate that *S. aureus* internalized macrophages, and intracellular *S*. *aureus* induced pyroptosis in professional phagocytic cells. Furthermore, inactive mTORC1 did not affect the ability of macrophages to phagocytose bacteria, our data may reveal the biological role of pyroptosis in *S. aureus* invasion.

In general, plasma membrane damage can be repaired efficiently in macrophages, for which endocytosis, membrane patching, and extracellular budding can be used [50]. Once gasdermin pores are present in numbers that exceed the cell’s compensatory abilities, the cells swell and the plasma membrane separates from the cytoskeleton in large fluid-filled bubbles and cell lytic dying [39]. In the present study, GSDMD-NT pore, swelling, and membrane rupture during pyroptosis—were observed. Further, our video showed pyroptosic cells swelling with characteristic large bubbles and eventually lysing with rapamycin or Stattic treatment. This morphological evidence demonstrates that *S. aureus* infection, mTORC1 inhibition, and STAT3 inhibition induce macrophage pyroptosis.

In our previous work, mTORC1 signaling was initiated by peptidoglycan (PGN) from *S. aureus* in mouse macrophages [51]. In the present study, we treated *S. aureus-*infected macrophages with rapamycin to inhibit mTORC1 and examine how *S. aureus*-induced pyroptosis is altered. We were surprised to find that *S. aureus*-induced pyroptosis was promoted by rapamycin in macrophages, for which there are 2 possibilities: rapamycin enhances *S. aureus* to induce pyroptosis and mTORC1 inhibition causes pyroptosis, in which case mTORC1 inactivation is an independent factor for macrophage pyroptosis. In rapamycin-treated cells, mTORC1 inactivation upregulated pyroptosic proteins, including CASP1 and GSDMD, release of IL-1β*,* and induced macrophage pyroptosis. Several studies in cells of mice and human reported that the negative regulation of inflammasome by rapamycin. Muhamuda et al. (2017) showed that rapamycin treatment (10 μM, 24 h) of lethal *Ixodes ovatus ehrlichia* (IOE) -infected WT-BMM attenuated production of IL-1β, which is the gold standard readout of inflammasome activity [52]. In addition, another group have observed that mTORC1 signaling inhibition by rapamycin (100 nM, 24 h) suppressed IL-1β secretion in these cells monocyte-derived macrophages (MDMs) [53]. In contrast, Rojas Márquez et al. [54] showed that mTORC1 inhibition by rapamycin (100 nM, 90 min) upregulation of IL-1β production in macrophages; Chimin et al. [55] reported that adipocyte mTORC1 deficiency promotes adipose tissue inflammation and NLRP3 inflammasome activation in mice with raptor deletion, which are in line with our findings of mTORC1 inhibition by rapamycin (100 nM, 6 h) upregulation of IL-1β and caspase-1 productions in the present study.

As a latent transcription factor, STAT3 modulates a range of target genes, including induced and repressed target genes that mediate cellular and organismal functions [56]. Moreover, STAT3 can be activated by LPS in human monocytes [30] and by PGN in mouse macrophages [51]. STAT3 regulates programmed cell death. Constitutive STAT3 activation leads directly to the induction of BCL-X, which inhibits of apoptosis [25]. STAT3 executes anti-autophagic functions by upregulating negative regulators of autophagy, such as MCL1, PIK3R1/p55a, and PIK3R1/p50a [26], and by downregulating essential autophagy genes, such as *BECN1* and *PIK3C3* [57]. Moreover, STAT3 negatively regulates gene expression in MEFs and cancer lines to control type I IFN-mediated antiviral response [58–60]. In fact, a whole-transcriptome profiling study showed that STAT3 acts as a transcriptional activator and suppressor, with a comparable number of upregulated and downregulated genes in diffuse large B cell lymphoma (DLBCL) cells [61]. In our cases, STAT3 was inhibited by the selective inhibitor Stattic, resulting in the upregulation of pyroptosic genes, including *CASP1*, *GSDMD* and triggering pyroptosis in human macrophages.

The nuclear localization of STAT3 comprises phosphorylation, dimerization, and nuclear localization, can be blocked by Stattic [62, 63]. In addition, phosphorylation of the Ser727 residue in the carboxyl transactivation domain might positively regulate STAT3 transcriptional activation; Dodd et al. demonstrated that STAT3 is phosphorylated directly by mTORC1 on Ser727 [64–66], confirming that mTORC1 functions in STAT3 activation. In a previous study, we found that rapamycin inhibits STAT3 phosphorylation [67], indicating that mTORC1 can regulate STAT3 activation. In the present study, mTORC1 was inhibited by rapamycin, accompanied by STAT3 inactivation and upregulation of pyroptosic gene. In the regulation of pyroptosis, STAT3 consistently had inhibitory functions, in accordance with the function of mTORC1 in pyroptosis in human macrophages. Further, we found that rapamycin prevents the nuclear localization of STAT3. Thus, mTORC1 combines with STAT3 to form the mTORC1/STAT3 axis to control pyroptosis in human macrophages.

## Conclusion

In summary, mTORC1 and STAT3 regulate human macrophage pyroptosis. The mTORC1/STAT3 axis is critical in pyroptosis in human macrophages. *S. aureus* invaded macrophages to induce pyroptosis; this process was promoted by rapamycin. mTORC1 regulates the expression of pyroptosic genes, including *CASP1* and *GSDMD*, through STAT3. Inhibition of mTORC1 prevents the nuclear localization of STAT3. The pathogenic effects of the inhibition of the mTORC1/STAT3 axis facilitates *S. aureus-*induced pyroptosis. This study demonstrates a regulatory function of the mTORC1/STAT3 axis in macrophage pyroptosis, constituting a novel mechanism by which pyroptosis is regulated in macrophages.

## Supplementary information

**Additional file 1: Figure S1.** PMA-induced differentiation of THP-1 to macrophages and further induction by LPS and IFN-γ into M1-polarized macrophages. THP-1 cells were treated with PMA (100 ng/mL) for 24 h to differentiate into Mϕ -polarized macrophages and then stimulated with IFN-γ (2.5 ng/mL) and LPS (100 ng/mL) to polarize them into classical M1 macrophages. (a) THP-1 and THP-1-derived macrophages and Mϕ and M1 macrophages. Scale bars represent 100 μm. (b) TFN-α and IL-1β in culture medium were determined by ELISA. M1 macrophages had more TFN-α and IL-1β than Mϕ macrophages. **p < 0.01. n = 3 independent experiments. Error bar indicates SD.

**Additional file 2: Table S1.** Number of *S. aureus* in macrophages.

**Additional file 3: Figure S2. ** (a) The amount of α-hemolysin in culture media and intracellular were evaluated by ELISA. (b) Effect of Cytochalasin B on macrophage phagocytosis. (c) Immunohistochemical analysis of pyroptosic protein GSDMD-NT in *S. aureus*-infected human macrophages. Scale bars represent 10 μm. (d) Large bubbles observed by light microscopy in *S. aureus*-infected macrophages. Large bubbles indicated by red arrows in pyroptotic cells. Scale bars represent 1 μm. *p < 0.05; **p < 0.01; NS = *p* > 0.05. n = 3 independent experiments. Error bar indicates SD.

**Additional file 4: Video 1.** The Live/Dead assay was performed by simultaneously monitoring the fluorescence after macrophages were infected with *S. aureus* for 3 h (MOI 25:1), Green, as live cell indicator and red, as dead cell indicator.

**Additional file 5: Figure S3.** (a) Immunohistochemical analysis of GSDMD-NT in human macrophages. Macrophages were preincubated with rapamycin for 6 h and then infected with *S. aureus*. Scale bars represent 10 μm. (b) Quantification of the number of CFU/ml of *S. aureus* in cells treated with rapamycin and without rapamycin. NS = *p* > 0.05. n = 3 independent experiments. Error bar indicates SD.

**Additional file 6: Figure S4.** Rapamycin inhibits mTORC1 signaling and upregulates GSDMD-NT expression. (a) Macrophages were treated with 100 nM rapamycin for 6 h. (b) Rapamycin upregulates GSDMD-NT in macrophages. Scale bars represent 10 μm. The resolved bands were quantified using Gel-Pro Analyzer 4.0 (Media Cybernetics, Inc., Rockville, MD, USA). *p < 0.05; **p < 0.01. n = 3 independent experiments. Error bar indicates SD.

**Additional file 7: Video 2.** Macrophages were treated with 100 nM rapamycin for 6 h, and pyroptosis was filmed in macrophages by laser confocal microscopy. A representative field was recorded. Arrow indicates lysing dead cell.

**Additional file 8: Video 3.** Macrophages were treated with 100 μM Stattic for 1 h, and pseudopod contracting, cell rounding, and lysing dead cells were filmed in macrophages by laser confocal microscopy. A representative field was recorded. Arrow indicate pseudopod contracting, cell rounding, and lysing dead cells.

**Additional file 9: Video 4.** Macrophages were treated with 100 μM Stattic for 1 h, and pyroptosis was filmed in macrophages by laser confocal microscopy. A representative field was recorded. Arrow indicate lysing dead cells.

## Data Availability

The datasets supporting the conclusions of this article are included within the article and its additional files.

## Abbreviations

MDMs: Monocyte-derived macrophages
THP-1: Human myeloid leukemia mononuclear
M1: Classically activated macrophages
mTORC1: Mammalian target of rapamycin complex 1
STAT3: Signal transducer and activator of transcription 3
PMA: Phorbol myristate acetate
IFN-γ: Interferon-γ
LPS: Lipopolysaccharide
*S*. *aureus*: *Staphylococcus aureus*
GSDMD: Gasdermin D
caspase-1: Cysteine-requiring aspartate protease 1
S6: Ribosomal protein S6
4EBP1: The eukaryotic initiation factor 4E binding protein 1
β-actin: Actin beta
IL-1β: Interleukin-1 beta
IL-18: Interleukin-18
NLRP3: NLR family pyrin domain containing 3
