# Supplementary material for: Pathogenic effects of inhibition of mTORC1/STAT3 axis facilitates Staphylococcus aureus-induced pyroptosis in human macrophages
Source: Cell Commun Signal. 2020 Nov 30;18:187. doi: 10.1186/s12964-020-00677-9 (PMC7706204; doi:10.1186/s12964-020-00677-9)
Supplement: Supplementary file 3 — Additional file 2: Table S1. Number of S. aureus in macrophages. [file 12964_2020_677_MOESM3_ESM.docx]

Table S1 Number of *S. aureus* in macrophages ($\bar{\text{X}}$±SD, n=3)

| Infection time | | 3 h |
| --- | --- | --- |
| *S. aureus*  (CFU/ml) | cell medium | 0  (7.5±0.12) ×10^3^ |
|  | whole cell lysate |  |
